# Including the Reason for Use on Prescriptions Sent to Pharmacists: Scoping Review

**DOI:** 10.2196/22325

**Published:** 2021-11-25

**Authors:** Kathryn Mercer, Caitlin Carter, Catherine Burns, Ryan Tennant, Lisa Guirguis, Kelly Grindrod

**Affiliations:** 1 Systems Design Engineering University of Waterloo Waterloo, ON Canada; 2 Library University of Waterloo Waterloo, ON Canada; 3 School of Pharmacy University of Waterloo Waterloo, ON Canada; 4 Advanced Interface Design Lab Systems Design Engineering University of Waterloo Waterloo, ON Canada; 5 Faculty of Pharmacy and Pharmaceutical Sciences University of Alberta Edmonton, AB Canada

**Keywords:** patient safety, human factors, patient engagement, multidisciplinary

## Abstract

**Background:**

In North America, although pharmacists are obligated to ensure prescribed medications are appropriate, information about a patient’s reason for use is not a required component of a legal prescription. The benefits of prescribers including the reason for use on prescriptions is evident in the current literature. However, it is not standard practice to share this information with pharmacists.

**Objective:**

Our aim was to characterize the research on how including the reason for use on a prescription impacts pharmacists.

**Methods:**

We performed an interdisciplinary scoping review, searching literature in the fields of health care, informatics, and engineering. The following databases were searched between December 2018 and January 2019: PubMed, Institute of Electrical and Electronics Engineers (IEEE), Association for Computing Machinery (ACM), International Pharmaceutical Abstracts (IPA), and EMBASE.

**Results:**

A total of 3912 potentially relevant articles were identified, with 9 papers meeting the inclusion criteria. The studies used different terminology (eg, indication, reason for use) and a wide variety of study methodologies, including prospective and retrospective observational studies, randomized controlled trials, and qualitative interviews and focus groups. The results suggest that including the reason for use on a prescription can help the pharmacist catch more errors, reduce the need to contact prescribers, support patient counseling, impact communication, and improve patient safety. Reasons that may prevent prescribers from adding the reason for use information are concerns about workflow and patient privacy.

**Conclusions:**

More research is needed to understand how the reason for use information should be provided to pharmacists. In the limited literature to date, there is a consensus that the addition of this information to prescriptions benefits patient safety and enables pharmacists to be more effective. Future research should use an implementation science or theory-based approach to improve prescriber buy-in and, consequently, adoption.

## Introduction

Medications are generally prescribed for conditions and illnesses for 4 reasons: to cure, to prevent, to slow progression, or to manage symptoms. Drugs can also be prescribed to help diagnose or manage the adverse effects caused by another medication or treatment, often referred to as *off-label* use. Sometimes the reason for use is apparent, such as using oral isotretinoin to treat nodular acne. Other times the reason for use is less apparent, such as using a hypertension medication to treat nightmares related to posttraumatic stress disorder [1].

To fill in the gaps when the reason for use information is not accessible, pharmacists must often rely on the patients to provide the reason for use information [2,3]. Yet, the accuracy of patient’s self-reported diagnosis varies widely. While the accuracy is quite good with conditions such as diabetes, it is very low for conditions such as rheumatoid arthritis or heart failure [4-6]. People who have difficulty communicating their diagnoses tend to be older, live with more chronic illness, and have a higher risk of death [7]. This puts the onus on the patient to correctly share the physician’s prescribing rationale and amplifies the risk for more vulnerable patients.

In the patient safety literature, there appears to be a consensus that it is safer for pharmacists to have access to a prescription’s reason for use [8]. While 80% of hospitals in the United States that have adopted some form of an electronic health record allow pharmacists to interact with the system to view laboratory tests and diagnoses, the reason for use is not identified as a core measure included in the electronic health record [7]. ePrescribing has facilitated the accuracy of prescriptions and some discussion with systems; however, many jurisdictions, including Canada, have not yet adopted this technology due to legislative or cost issues. Therefore, while pharmacists may have access to the patient’s health information used by the prescriber to determine the reason for use, they must infer the reason without its explicit inclusion on the patient’s record. In contrast to community pharmacies where access to national electronic health record data is only available in some countries and regions, communication of the reason for use remains both a desire of pharmacists and a challenge for health care systems [3,8,9].

Most prescriptions today are written electronically [10]. With the potential for timely access by prescribers and pharmacists, digital prescription records could support the communication of a prescription’s reason for use along with the right design. Schiff et al [10] tested an indication-based prescribing system that makes it easier for prescribers to share a prescription’s reason for use. In their electronic prescribing system, prescribers start with a diagnosis or problem and then select a treatment option from a list of recommendations. The system would additionally provide suggestions based on a patient’s health history, but still allow for complete autonomy of a prescribers’ selection [10]. However, there still appears to be very little information on how to include a prescription’s reason for use to support pharmacist’s decision making.

The objective of this interdisciplinary scoping review is to characterize the research on how including the reason for use on a prescription impacts pharmacists. Given that this topic spans multiple disciplines, the first step is to map relevant literature to identify the potential size and scope. Our goals were to describe the research on the design, implementation, and evaluation of the reason for use information for pharmacists, including the types and sources of evidence, and the areas where further research is needed. When literature on a particular topic is scattered through different disciplines, there is a real risk that the research will be siloed and will not reach those who are in a position to translate the research into practice. Thus, we also aimed to provide health care, informatics, and engineering researchers with a cohesive summary of the reason for use studies to date, as it relates to pharmacists.

## Methods

### Study Framework

We followed the scoping review framework developed by Arksey and O’Malley [11], and conducted the reporting using the PRISMA Extension for Scoping Reviews (PRISMA-ScR) Checklist [12]. We carried out the following 5 stages of a scoping review: (1) identify the research question, (2) identify relevant studies, (3) select articles, (4) chart the data, and (5) collate and summarize the data [13]. To build the search strategy, we used the SPIDER tool (sample, phenomenon of interest, design, evaluation, research type) to identify qualitative and mixed method studies [14]. We also used the traditional PICO tool (patient, intervention, comparator, outcome) to develop a search strategy for quantitative studies, such as randomized controlled trials [15].

### Information Sources

We searched the following databases for journal articles and conference proceedings between December 2018 and March 2019, and ran an update in January 2019: PubMed, Institute of Electrical and Electronics Engineers (IEEE), Association for Computing Machinery (ACM), International Pharmaceutical Abstracts (IPA), and EMBASE. Searches were conducted between December 2018 and January 2019. We also hand-searched reference lists from relevant articles. We exported all search results to EndNote reference manager software (version 8; Clarivate Analytics) and removed duplicates. The EndNote File was exported to Covidence (Veritas Health Innovation Ltd.).

### Search

Three librarians worked together to build a comprehensive search strategy for each database, with support from database specialists. We began by familiarizing ourselves with the terminology for “reason for use” by conducting a preliminary search on PubMed and by searching reference lists of known publications on the topic. Developing a search strategy for each database was complex, balancing the need to be as comprehensive as possible while limiting the noise caused by the wide-reaching “indication” search term. Detailed search strategies are presented in Multimedia Appendix 1. A sample search strategy for PubMed is as follows:

((“reason for use”[All Fields] OR Indication*[All Fields] OR Off-Label Use[MeSH terms] OR (diagnosis[All Fields] OR diagnosis[MeSH terms] AND (pharmacists[MeSH Terms] OR pharmacist*[All Fields])) AND (prescription[All Fields] OR drug prescriptions[MeSH Terms] OR prescriptions[MeSH Terms]) AND (documentation[MeSH Terms] OR document[All Fields] OR record[All Fields] OR communication [MeSH terms] OR communication[All Fields] OR Electronic health record[MeSH Terms] OR “electronic medical record” OR labels[All Fields] OR off-label[All Fields] OR Off-Label Use[MeSH Terms] OR electronic prescribing[MeSH Terms]) AND (collaboration OR intersectoral collaboration[MeSH Terms] OR interprofessional relations[MeSH Terms] OR patient care team[MeSH Terms] OR professional role[MeSH Terms] OR team[All Fields] OR interprofessional[All Fields] OR “interprofessional collaboration” [All Fields] OR patient[All Terms] OR patients[MeSH Terms]))).

### Selection of Sources

We imputed titles and abstracts into Covidence and 2 authors (KM and CC) independently screened the titles, abstracts, and full-text articles according to the eligibility criteria. Studies were eligible for inclusion if they included pharmacists as part of the study and examined one of the following: (1) the inclusion of reason for use in a prescription; (2) the addition of reason for use to a prescription medication label; or (3) why prescribers do or do not include reason for use in prescriptions. We did not limit ourselves to a specific type of study, or field of study. We did not place any limits on the date or location of publications other than the research must be published in English. We excluded dissertations and commentaries.

### Data Synthesis

One researcher used a standardized form to extract data from included full-text articles, and the data were verified by a second researcher. We recorded the following data: lead author, year of publication, geographic location, participants, methods, analysis, research setting, outcomes, and location of the reason for use (eg, electronic health record, written prescription). While reviewing the included articles, we were guided by the research question for this study: “How are pharmacists affected when the reason for use is included on a prescription, and what are its implications for collaboration and patient safety.” We began by categorizing the literature according to the methodology, key findings, and setting. As we reviewed the articles, we added categories as necessary to understand the full extent of themes and research currently being carried out. We identified gaps and key findings after reviewing the final list of included articles.

## Results

### Study Selection

**Figure 1:**
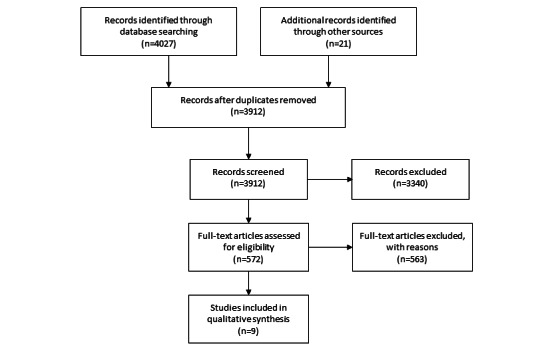
PRISMA (Preferred Reporting Items for Systematic Reviews and Meta-analyses) diagram.

We identified a total of 4027 titles with an additional 21 studies identified from other sources (Figure 1), of which 136 were duplicates. After screening, 3912 articles were screened, leaving a total of 9 that met the inclusion criteria [2,16-23] (Multimedia Appendix 2). Examples of reasons papers were excluded included the following: focus on labeling not prescriptions [24,25], did not include a pharmacist [26-30], focused on medication review without indication [31,32], monitoring drug treatment [33], and network data mining [2,16-23,34].

The literature was synthesized into 4 areas of focus: (1) terminology (2) importance of including reason for use on prescriptions; (3) impact of reason for use on decision making and workflow; and (4) barriers to reason for use information.

### Descriptive Characteristics

The 9 included studies were published between 1998 and 2018. Six studies examined pharmacists and physicians jointly in the studies [16-18,21-23]. In total, 4 studies were conducted in the United States [2,18,21,23], 2 in Europe [20,22], 1 in the Middle East [16], and 2 in Australia [17,19]. Two studies focused on prescribing in hospital [16,22], 6 focused on primary care [2,17,19-21,23], and 1 involved a consultation with experts from different settings [18]. According to the inclusion criteria, all studies included pharmacists, with 7 also including physicians [16-19,21-23], 4 included patients [18,21-23], and 1 presented results from a pilot study with various stakeholders [18]. Five studies used a qualitative approach to capture perspectives on the application of reason for use [17,18,20,21,23] and 4 used a quantitative approach to characterize the impact of reason for use [2,16,19,22]. Three of the included studies were published in health research journals [17,19,22], with the remaining 6 published in pharmacy practice journals [2,16,18,20,21,23]. We did not identify any studies in the engineering or informatics literature.

### Terminology

Including a reason for use on a prescription was described in a variety of ways. The most common terminology is “indication” [17,18,20] or related terms including “indication in prescription” [16], “medication indication [21], and “indication for treatment” [22]. Other terminologies were patient diagnosis [23], “reason for use” [2], purpose of the medication” [19], and “clinical patient data” [35].

### Current Perspectives on Including Reason for Use on Prescriptions

All included studies identified that reason for use is needed to improve patient safety. Generally, the pharmacist and physician research participants had positive reactions toward adding the reason for use to prescriptions. Using semistructured interviews with pharmacists, physicians, and patients, Garada et al [17] identified that the addition of reason for use information can reduce perceived prescribing and dispensing errors, and that adding the information to the label supports patient engagement and the work of other health care professionals. Liddell and Goldman [19] specifically identified that including the reason for general use was the most important aspect of new prescription notations to improve communication.

### Impact of Reason for Use on Decision Making and Workflow

Three studies mentioned pharmacists feeling limited by missing information [20,21,23], 3 identified the reason for use as being important to pharmacists for catching prescribing errors and improving safety [16,17,22], 4 recognized the potential for reason for use information to improve workflow [16,18,21,23], and 3 discussed the need for reason for use to provide accurate patient counseling [21,23,35]. Of the 3 studies that examined workflow, Al-Khani et al [16] identified the difficulty in getting physicians to comply with including reason for use, and the subsequent change in workflow.

Al-Khani et al’s [16] study used a hospital’s safety reporting system to show that 35% of the drug prescribing errors that pharmacists flagged were identified using reason for use. Liddell and Goldman [19] demonstrated a very positive response from physicians about being more collaborative with pharmacists when notations were included that specify the reason for use information, and both pharmacists and physicians were positive about tools that would facilitate communication.

Improved collaboration and communication between pharmacists and physicians were identified in 2 articles [20,21]. Tarn et al [21] identified the potential benefit that improved collaboration can have on efficiency. Kron et al [18] discussed how pharmacists often try to infer information about why a medication was prescribed from the patients, which is supported by Warholak et al’s [23] findings that after a diagnosis was included on an electronic prescription, pharmacists have less confusion and uncertainty [18,23], further identifying that patients are used as an intermediary to get access to information.

### Barriers to the Reason for Use Information

Only 1 paper examined privacy concerns, concluding that while pharmacists and physicians were concerned about privacy, patients were not generally concerned with the privacy implications of documenting reason for use on a prescription [17]. Of the 5 included studies that mentioned technology [16-18,22,23], 4 suggested there was a need to improve the prescribing software available [17,18,22,23]. Four studies examined electronic prescribing [16-18,23].

Raebel et al [35] discussed the effectiveness of a computerized pharmacy alert system and active collaboration between health care professionals. The study’s goal was to improve prescribing safety and identified that a barrier to this was that clinical patient data were not easily available to many pharmacists [35]. Kron et al [18] specifically examined the difficulties in encouraging prescribers to include the reason for the prescription, and identified that electronic prescribing was laying the foundation for future adoption.

## Discussion

### Principal Findings

We set out to identify and describe the current literature around how the reason for use information can be shared with a pharmacist through a prescription. We identified several studies where systems supported a mandatory reason for use field or modified the computer interface to make it easier for prescribers to add this information. The research to date has not moved much beyond the typical barriers to adoption such as a lack of time or incentives; however, when asked, prescribers do clearly identify the benefits of adding this information. Therefore, one of the key findings of this review is we did not identify any implementation science or theory-based studies aiming to improve adoption by prescribers.

One observation from this study was that the problem of sharing reason for use information is greater than making the reason for use field mandatory for prescribers. The study by Al-Khani et al [16] in a hospital in Saudi Arabia retrospectively identified that pharmacists’ access to the reason for use and medication history was a major factor in identifying up to 60% of errors, even though some physicians found ways to override the mandatory field on prescriptions by writing characters or letters. Through their experiences in a Chinese hospital, Li and Zhou [36] also highlighted that a hospital-wide policy could promote the addition of reason for use to electronic prescriptions—allowing the pharmacy department to keep the proportion of “inappropriate physician orders” below 1%—but that it raised a new challenge when prescribers provide poor quality or incomplete information [36]. Another study in a Dutch children’s hospital also found that prescribers rejected up to half of pharmacist recommendations due to a lack of timeliness or relevance—highlighting that improved information around indication would lead to more timely and meaningful collaboration between prescribers and pharmacists [37]. Moving forward, researchers could benefit from using an implementation science framework to look at the reasons interventions or policies worked (or not), especially in critical areas related to reach, effectiveness, adoption, implementation, and maintenance over the long term [38].

One major limitation of this review was that the terminology used to describe “reason for use” was not consistent, which is a barrier to building an evidence-based body of knowledge to encourage designing, implementing, and ultimately having an uptake of including a reason for use on prescriptions. If the language used is not consistent, it is difficult to make sure all stakeholders are working on the same problem, toward the same solution. The reason for use literature bridges health, engineering, informatics, and other areas, all with different terminologies, frameworks, and methods. The papers included in this study were all from health care journals, primarily pharmacy journals. This may mean that engineering and informatics disciplines are not aware of these papers. While the methodology for health-related scoping reviews is well documented [11], the search methodology and available tools have not yet caught up in other disciplines. For example, while PubMed uses the MeSH search terms and EMBASE uses Emtree, these are not standard between databases, and the nonmedical databases do not have standardized search terms.

### Conclusions

In the limited literature to date, there is a consensus that the addition of reason for use information to prescriptions benefits patient safety and enables pharmacists to be more effective. However, it is also clear that very little has been done to motivate prescribers to include this information, despite clear benefits such as reducing the number of phone calls received by pharmacists. Future research should be multidisciplinary, and use an implementation science or theory-based approach to improve prescriber buy-in and, consequently, adoption.

## Appendix

Multimedia Appendix 1 Final searches.

Multimedia Appendix 2 Included studies.
