# Supplementary material for: Including the Reason for Use on Prescriptions Sent to Pharmacists: Scoping Review
Source: JMIR Hum Factors. 2021 Nov 25;8(4):e22325. doi: 10.2196/22325 (PMC8663503; doi:10.2196/22325)
Supplement: Multimedia Appendix 1 [file humanfactors_v8i4e22325_app1.pdf]

Appendix 1: Final searches

PubMed:

(("reason for use"[All Fields] OR Indication*[All Fields] OR Off-Label Use[MeSH terms] OR (diagnosis[All Fields] OR diagnosis[MeSH terms] AND (pharmacists[MeSH Terms] OR pharmacist*[All Fields])) AND (prescription[All Fields] OR drug prescriptions[MeSH Terms] OR prescriptions[MeSH Terms]) AND (documentation[MeSH Terms] OR document[All Fields] OR record[All Fields] OR communication [MeSH terms] OR communication[All Fields] OR Electronic health record[MeSH Terms] OR “electronic medical record” OR labels[All Fields] OR off-label[All Fields] OR Off-Label Use[MeSH Terms] OR electronic prescribing[MeSH Terms]) AND (collaboration OR intersectoral collaboration[MeSH Terms] OR interprofessional relations[MeSH Terms] OR patient care team[MeSH Terms] OR professional role[MeSH Terms] OR team[All Fields] OR interprofessional[All Fields] OR “interprofessional collaboration” [All Fields] OR patient[All Terms] OR patients[MeSH Terms]))) 1610

IPA

1	("reason for use" or Indication or indications).mp.
2	(diagnosis and (pharmacists or pharmacist)).mp.
3	(prescription or drug prescriptions or pharmaceutical preparation).mp.
4	(documentation or document or record or communication or Electronic health record or electronic medical record or labels or off-label or electronic prescribing).mp.
5	(collaboration or interprofessional relations or professional role or team or interprofessional or interprofessional collaboration or patient).mp.
6	1 or 2
7	3 and 4 and 5 and 6
#	89 total results

Embase

1	exp treatment indication/ or exp drug indication/
2	"reason for use".mp.
3	exp diagnosis/ae [Adverse Drug Reaction]
4	exp non prescription drug/ or exp prescription/ or exp prescription drug/
5	exp empowerment/ or exp follow up/ or exp document delivery/
6	information processing/ae [Adverse Drug Reaction]
7	exp hospital information system/ or exp medical record/ or exp information system/ or exp electronic medical record/ or exp "organization and management"/ or exp computer system/ or exp computer/
8	exp prescription/ or exp "off label drug use"/ or exp "drug use"/ or exp drug indication/ or exp drug labeling/
9	exp patient care/ or collaboration.mp. or exp cooperation/ or exp intersectoral collaboration/

10	exp health care personnel/ or exp professional practice/ or interprofessional.mp.
**11**	**1 or 2**
12	5 or 6 or 7
13	3 or 4 or 8
14	9 or 10
15	11 and 12 and 13 and 14

1513

ACM

(("reason for use" OR Indication) AND (document OR documentation OR record OR label) AND (prescription OR drug OR medic*)) 195

IEEE:

(("reason for use" OR Indication OR (diagnosis AND pharmacist)) AND (prescription OR pharmaceutical OR drug OR medic*) AND (documentation OR document OR record OR communication OR labels OR off-label OR electronic prescribing)) (218)
