# Supplementary material for: Including the Reason for Use on Prescriptions Sent to Pharmacists: Scoping Review
Source: JMIR Hum Factors. 2021 Nov 25;8(4):e22325. doi: 10.2196/22325 (PMC8663503; doi:10.2196/22325)
Supplement: Multimedia Appendix 2 [file humanfactors_v8i4e22325_app2.pdf]

Table 1: Included Studies

**Authors**	**Year**	**Country**	**Objective**	**Location of Pharmacist**	**Study Type**	**Reason for Use Format**	**Outcome Related to Pharmacists and Reason for Use**
Al-Khani[25]	2014	Saudi Arabia	To explore factors that help pharmacists identify and thus prevent harm from incorrect drug prescribing errors in an ambulatory care setting.	Hospital	Retrospective analysis of pharmacist-identified errors	Mandatory reason for use field on a hospital CPOE system	Reviewing the mandatory indication field on prescriptions helped pharmacists identify 35% of all prescribing errors that were voluntarily reported
Garada[26]	2017	Australia	To investigate the perspectives of Australian consumers, pharmacists and prescribers on documenting the indication on prescriptions and dispensed medicines labels.	Community Pharmacy	Semi-structured interviews of patients, physicians, and pharmacists	Mock-ups of dispensed medication labels including reason for use	Participants felt that adding reason for use to the label would help pharmacists identify look-alike/sound-alike errors, especially when the drug has multiple indications. Pharmacists preferred a specific reason for use (urinary tract infection) over a general one (infection).

Kron[7]	2018	United States	To consult high-level stakeholders on system design considerations and requirements necessary for building and implementing an indications-based CPOE system.	Expert Panel	Stakeholder panels	--	Reason for use needs to be incorporated into prescriber workflow, such as through an indication-based prescribing CPOE system.
Liddell	1998	Australia	To evaluate the rate of use and acceptance of a new prescription form designed to provide more information to pharmacists and patients	Community	Education session and trial new prescription forms	New prescription forms	Most GPs and pharmacists saw indicating the purpose of the medication on the prescription as the most substantial and important focus of the new prescription notations.
Stakenborg[28]	2016	Netherlands	To describe pharmacists' and pharmacy assistants' experiences with parents contacting the pharmacy for a febrile child, and to identify ways of improving medication	Community Pharmacy	Focus Groups	--	Antibiotic prescriptions were more common after hours. Pharmacy staff felt the reason for use is most important when doses are too low, thereby allowing pharmacists to double check the

			management of these children.				dose, and improve medication safety. Some pharmacies already had agreements with physicians to include the reason for use and for pharmacists to automatically correct a dose if it was too low.
Tarn[29]	2012	United States	To investigate older patient, physician and pharmacist perspectives about the pharmacists' role in pharmacist-patient interactions.	Community	Focus Group	--	The "barriers to communication" theme identified that pharmacists felt discussions with patients were limited because they did not have the reason for use information.
Vercheval[30]	2016	Belgium	To improve the quality of documentation on antibiotic therapy in the computerized medical records of inpatients.	Hospital	Prospective Interrupted Time Series	Reason for use recorded in prescriptions ordered through CPOE	A multifaceted intervention increased the documentation of antibiotic reason for use from 83% of prescriptions to 90% of prescriptions (p=0.0013)

Warholak-Juarez[2]	2000	United States	To evaluate the effect of incremental increases in patient information on the quality of pharmacists' clinical decisions related to legally mandated prospective drug utilization review responsibilities.	Community Pharmacy and Indian Health Services	Simulation	Reason for use added to prescriptions	Pharmacists identified drug therapy problems better when they had access to the patient's reason for use and this continued to improve as pharmacists had more experience.
Warholak[31]	2014	United States	To compare the incidence and types of potential drug therapy problems identified prior to and after providing the pharmacist with patient diagnosis information.	Community Health Centre	Prospective Pre-Post Study	Free-text reason for use added to electronic prescription	The pharmacist intervention rate decreased from 3.9% before the reason for use was added, to 1% after the reason for use was added ($p<0.001$).
